# A Pediatric Patient With Type 1 Diabetes Mellitus With Poor Glycemic Control, Medication-Resistant Hypertension, and New-Onset Headache Diagnosed With Adrenocorticotropic Hormone (ACTH)-Secreting Pituitary Macroadenoma

**DOI:** 10.7759/cureus.35698

**Published:** 2023-03-02

**Authors:** Megan Ingley, Kristen Schmidbauer, Patrick Quebedeaux, Idris Ali Amghaiab, Jacqueline T Chan

**Affiliations:** 1 Pediatric Medicine, Medical College of Georgia at Augusta University, Augusta, USA; 2 Pediatric Endocrinology, Medical College of Georgia at Augusta University, Augusta, USA

**Keywords:** pediatric hypertension, pediatric endocrinology, panhypopituitarism, hypercortisolemia, acth secreting tumor, hypercortisolism, pituitary adenoma, cushing’s disease, : diabetes mellitus

## Abstract

The most well-known cause of hyperglycemia is diabetes mellitus, a condition that affects the body's ability to either use (type 2 diabetes mellitus - T2DM) or produce (type 1 diabetes mellitus - T1DM) insulin. Exogenous insulin is the mainstay therapy to achieve optimal glucose control in T1DM, though glucose hemostasis is affected by several factors. Following the initiation of insulin therapy, symptoms of polyuria, polydipsia, and weight loss are reversed. Diabetes mellitus is associated with several complications, including but not limited to, renal disease (hypertension, microalbuminuria), peripheral neuropathy, delayed growth, and delayed puberty. Hyperglycemia can also be caused by acute illness, surgery, trauma, infection, parenteral nutrition, obesity, or other medical conditions such as Cushing syndrome and polycystic ovarian syndrome. While refractory hyperglycemia is often attributed to poor adherence to medications, other organic etiologies should also be considered, especially in the setting of early-onset complications of diabetes mellitus. In this report, we present a case of a pediatric patient with T1DM with refractory hyperglycemia and medication-resistant hypertension who was lost to follow-up. When he returned to the endocrinology clinic, he had Cushingoid features and a headache. After multiple admissions for hypertension, the patient was discovered to have a pituitary macroadenoma. Following the removal of the adenoma, the patient's insulin requirement decreased substantially and his blood pressure returned to normal, allowing all blood pressure medications to be discontinued.

## Introduction

Diabetes mellitus is the most common chronic disease in children, with type 1 diabetes accounting for 80% of diabetic patients under the age of 19 years [1]. Between 2002 and 2015, the incidence of type 1 diabetes mellitus (T1DM) in patients under 20 years of age in the United States increased by 1.9% with the rate of increase generally higher among African American, Hispanic, Asian, and Pacific Islander youths [2].

Patients with T1DM typically present to their primary care provider (PCP) with decreased activity levels, polydipsia, polyuria, and weight loss. Subsequent point-of-care testing reveals hyperglycemia with glucose and/or ketones in the urine. The second most common presentation of T1DM is diabetic ketoacidosis (DKA), which accounts for approximately 30% of initial presentation [3] and is the most common cause of hospitalization and mortality in young patients with diabetes [4]. Generally, these patients present to the emergency department with a characteristic fruity odor, increased respiratory rate (Kussmaul breathing) and decreased level of consciousness. There is typically a preceding history of polydipsia, polyuria, and weight loss.

Though hyperglycemia in pediatric patients with diabetes mellitus commonly occurs due to poor adherence to insulin therapy, other etiologies should be considered when hyperglycemia is refractory and additional systemic signs and symptoms are present. In this report, we discuss a case of a pediatric patient presenting with signs and symptoms of elevated cortisol levels several months after being diagnosed with T1DM.

## Case presentation

A pre-pubertal 11-year-old male presented to the emergency department (ED) with clinical symptoms and labs consistent with DKA. He was admitted to the pediatric intensive care unit (PICU) for initial management, where his HbA1c was measured to be 17.9%. Diabetes teaching was provided while inpatient and he was subsequently discharged on long-acting and short-acting insulin. Antibodies to glutamic acid decarboxylase 65-kilodalton isoform (GAD65) and islet cell antigen 2 (IA2) were positive, indicating that the patient had T1DM.

He followed up two weeks later at the endocrinology clinic where outpatient blood sugars were reported to range between 200 and 400 mg/dL, and insulin doses were adjusted accordingly. He failed to return to his one-month follow-up appointment but did come six months after his initial outpatient appointment and then one month later. His HbA1c was noted to be improving, though still elevated, at both visits (12.7% and 11.3%, respectively).

Eleven months after his initial diagnosis of T1DM, the patient was seen at the endocrinology clinic for a routine follow-up. His HbA1c was measured to be 10.7%. His blood pressure on arrival was noted to be 139/107 mmHg (>95th percentile for age and gender). At this visit, the family reported a one-month history of facial and abdominal swelling and noted that the family history was significant for thyroid disease. Since myxedema could not be ruled out, thyroid-stimulating hormone (TSH) and free T4 (fT4) levels were collected. TSH was low (0.214 mIU/mL) while fT4 was on the low end of normal (0.85 ng/dL). Hypertension and generalized edema raised concerns for underlying renal disease, such as nephrotic syndrome, and hence the patient was sent to pediatric nephrology who evaluated the patient later that day. Laboratory studies evaluating for liver and renal pathologies were unremarkable except for mildly elevated urine microalbumin. Lisinopril 5 mg daily was initiated, renal ultrasound was ordered, and the patient was instructed to follow up in one month.

At his next nephrology visit two months later, the patient's blood pressure had improved to 123/89 mmHg and lisinopril 5 mg was continued. Renal ultrasound revealed non-obstructing bilateral renal stones and he was referred to urology. Between this visit and his scheduled follow-up one month later, the patient called the office to report angioedema and cough. His medication was subsequently changed from lisinopril to amlodipine 10 mg daily.

**Figure 1:**
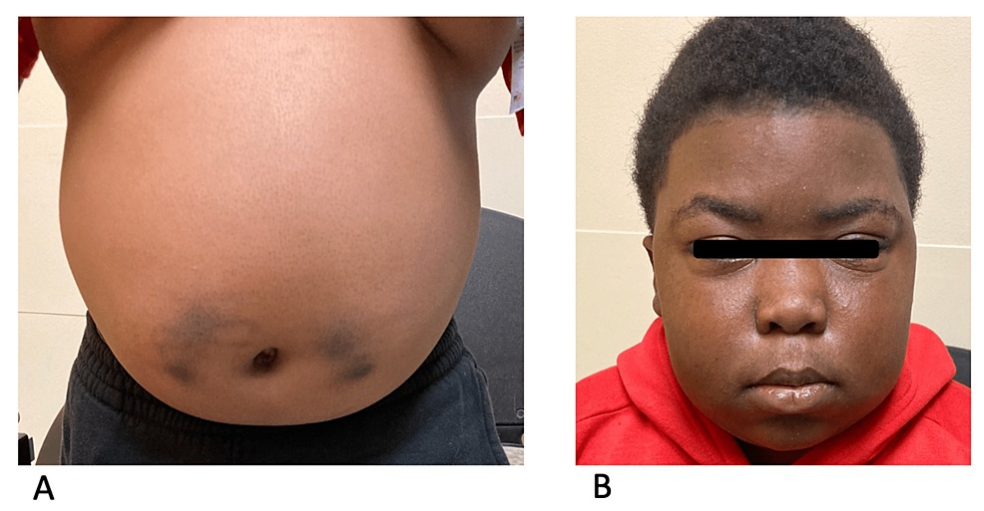
Physical findings 1A: distention and bruising of the abdomen; 1B: facial edema in "moon facies" distribution

Four months after his initial referral to nephrology, the patient presented to the ED with a chief complaint of testicular pain. The pain was controlled with morphine, but the patient's blood pressure was persistently elevated (up to 158/104 mmHg) even in the absence of pain. As such, the patient was admitted for pain control and blood pressure management. During this admission, pediatric nephrology was consulted, and carvedilol 25 mg twice daily was added to his anti-hypertensive therapy. The patient’s abdominal distention (Figure 1A) and facial edema (Figure 1B) had not improved (five months after onset), and hence the primary team ordered a random total cortisol level, which was elevated at 27.76 mcg/dL. At this time, it was decided that since the patient's blood pressure was now under control and he was otherwise medically ready for discharge, the elevated cortisol level would be investigated further in the outpatient setting. The patient was subsequently discharged with instructions to take amlodipine 10 mg daily and carvedilol 25 mg twice daily.

**Figure 2:**
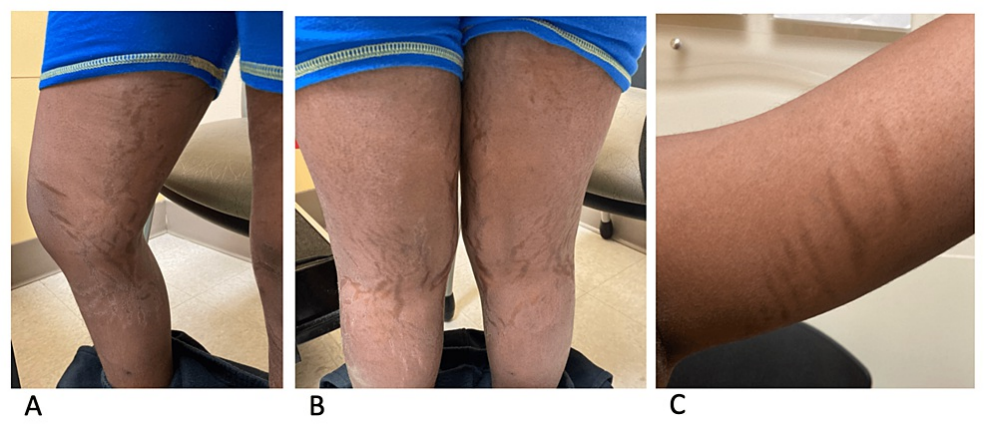
Physical features 2A and 2B: striae seen on posterior and medial aspects of bilateral thighs and legs; 2C: striae seen on the medial aspect of the left arm

One month later (six months after the onset of his swelling and hypertension), the patient went to his pediatrician for routine care. At this time, his physical exam was significant for moon facies, dark striae, bruising, and gynecomastia. In addition, he had a decline in growth velocity and significant weight gain. His blood pressure was noted to be elevated to 146/100 mmHg, and hence he was sent to the ED for further management. While in the ED, his blood pressure remained elevated despite multiple doses of nifedipine. Due to the inability to control his blood pressure with oral medications, the patient was admitted. During the admission process, the patient reported a one-week history of headaches. He reported that his home blood pressure monitor always showed that his blood pressure was high when he had a headache. It was also noted during this time that the patient and his family had misunderstood the instructions for his blood pressure medications and that while he had been taking carvedilol 25 mg twice daily, he had not been taking amlodipine 10 mg daily. Per pediatric nephrology recommendations, the patient was continued on his prescribed home anti-hypertensive regimen plus nifedipine 10 mg as needed while admitted. The following day, endocrinology was consulted due to new-onset violaceous striae on the patient's extremities(Figures 2A, 2B, 2C). They recommended initiating a workup to evaluate for Cushing syndrome. Various labs, including serum total cortisol, 24-hour urine-free cortisol, and adrenocorticotropic hormone (ACTH) levels were collected. The patient's headaches were closely monitored and appeared to correlate with higher blood pressure measurements. Headaches were well-managed with ibuprofen and acetaminophen throughout the day.

**Figure 3:**
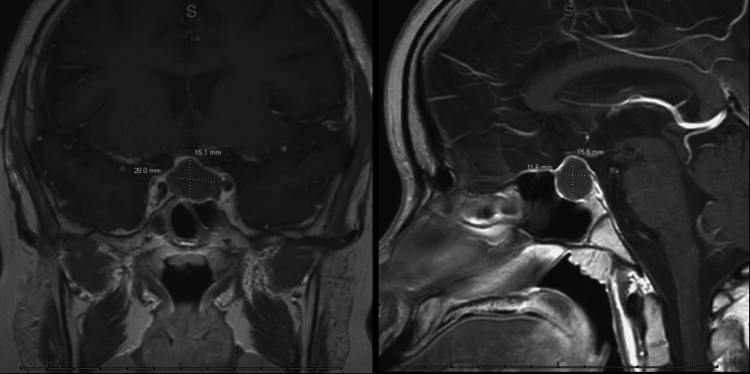
MRI brain showing pituitary macroadenoma MRI: magnetic resonance imaging

Overnight, he experienced the onset of a severe headache over the central and frontal region behind his eyes, which was different from his previous headaches. He also had associated symptoms of blurry vision and photophobia. His blood pressure was normal and the headache was no longer responding to acetaminophen and ibuprofen as it had throughout the day. Given his history, clinical presentation, and refractory headache, a non-contrast head CT was obtained to evaluate for an intracranial mass or acute intracranial hemorrhage. The CT revealed a pituitary mass without evidence of hemorrhage. A subsequent brain MRI showed a sellar mass measuring 14 x 21 x 16 mm, which was favored to represent a pituitary macroadenoma. There was no extension of the mass into the cavernous sinus, but mild deformation of the right optic chiasm, moderate supratentorial parenchymal volume loss, and a few scattered foci of FLAIR signal hyperintensities (Figure 3) were noted.

At this point, more labs were obtained to test for pituitary function (Table 1), The patient’s 24-hour urine-free cortisol and serum total cortisol collected earlier in the day were both elevated. Pediatric neurosurgery was consulted, and a joint transsphenoidal complete resection of the pituitary macroadenoma was performed with pediatric otolaryngology assistance. The final pathology report confirmed that the mass was a “pituitary adenoma with changes consistent with apoplexy.” The tumor was not stained for hormones. Prior to resection, serum total cortisol had normalized and hence stress-dose steroids were not provided.

**Table 1:** Lab values and interpretations GH: growth hormone; IGF: insulin-like growth factor; LH: luteinizing hormone; FSH: follicle-stimulating hormone; TSH: thyroid-stimulating hormone; PTH: parathyroid hormone; ACTH: adrenocorticotropic hormone

Lab test (reference range)	Before presumed apoplexy	After presumed apoplexy, prior to resection	Post-resection
GH (0.01-0.97 ng/mL)		9.71, high	
IGF-1 (79-506 ng/mL)		47, very low	72, low
IGF binding protein (2.4-8.4 mcg/mL)		3.7, normal	2.4, low normal
Prolactin (2.1-17.7 ng/mL)		1.36, normal	
LH (<0.1-6.0 mIU/mL)		<0.20, normal	
FSH (1.4-18.1 mIU/mL)		0.67, low	
TSH (0.4-4.7 mIU/mL)		0.214, low	1.766, normal
Free T4 (0.58-1.76 ng/dL)		0.85, normal	0.64, normal
ACTH (7.2-63 pg/mL)	Sample rejected	38, normal	
Serum total cortisol (3-22 mg/dL)	27.76, high	7.91, normal	0.71, low
Urine-free cortisol (2.6-37 mcg/24h)	1,304, very high (3,950 mL)	<1.2, very low (1,525 mL)	

**Figure 4:**
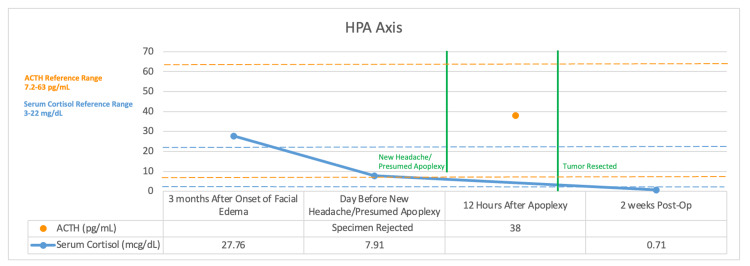
ACTH and cortisol levels ACTH: adrenocorticotropic hormone

Postoperative labs showed low cortisol (Figure 4) and low IGF-1. The patient was started on oral prednisone, titrated to effect, with plans to start growth hormone replacement in the future.

**Figure 5:**
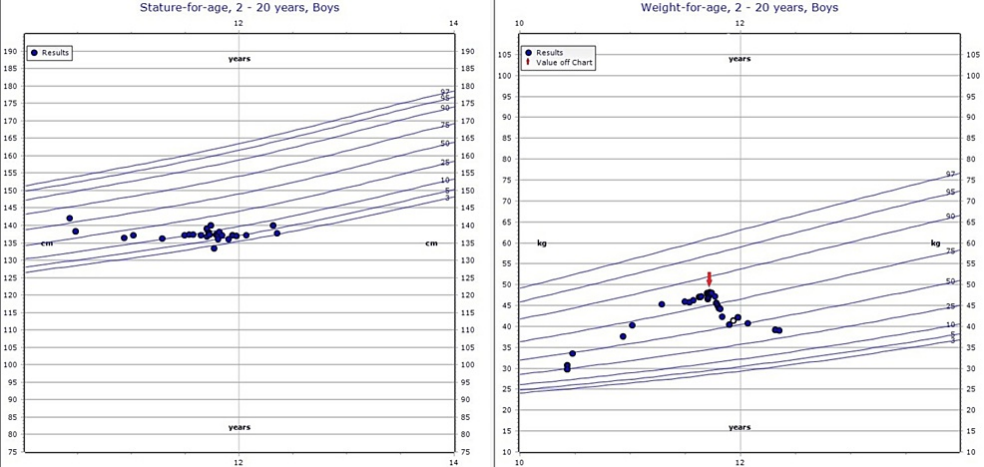
Stature- and weight-for-age charts: CDC The red arrow represents surgical resection CDC: Centers for Disease Control and Prevention

Following resection, the patient's hypertension resolved, and he no longer requires any antihypertensive medications. Additionally, his long-acting insulin requirement decreased by 50%. At his three-month postoperative follow-up, the patient’s striae and facial edema had improved significantly and his weight decreased by 25 pounds. The patient will continue to be followed up by endocrinology for T1DM as well as monitoring and treatment of hypopituitarism. Figure 5 depicts the Centers for Disease Control and Prevention (CDC) charts for stature- and weight-for-age

## Discussion

Unlike patients with type 1 diabetes who often present in DKA, patients with Cushing disease often present with non-specific signs and symptoms that manifest themselves over a prolonged period of time, making it difficult from a diagnostic perspective. Initial presentation commonly includes weight gain (90% of patients at presentation), growth failure (83%), hirsutism or acne (78%), amenorrhea and delayed puberty (78%), centripetal obesity such as buffalo hump or moon facies (75%), osteopenia (70%), violaceous striae (60%), hypertension (50%), headaches (25%), easy bruising (25%), compulsive behaviors and emotional lability (20%), and muscle weakness (12%) [5]. When presented separately, each of these problems has a broad differential diagnosis, but when considered together, they point toward a single etiology: hypercortisolism. Cushing syndrome is a rare entity with only 39.5-48.6 cases per million per year in Americans less than 65 years of age with the annual incidence of Cushing disease being significantly lower at 6.2-7.6 cases per million [6]. Furthermore, the annual incidence of Cushing disease in Americans less than or equal to seven years of age was 2.4-2.8 per million (females: 2.5-4.0 per million, males: 0.8-3.0 per million) as reported in 2014 [6]. The most common cause of Cushing syndrome is exogenous glucocorticoid administration, but once it is ruled out based on clinical history, endogenous causes should be investigated. The most common cause of endogenous Cushing syndrome in infants is primary adrenal tumors, whereas the most common cause of Cushing syndrome in children over 5 years of age is an ACTH-secreting pituitary adenoma [5].

Our patient initially presented in DKA without any additional signs or symptoms to suggest hypercortisolism. As the patient was lost to follow-up, the exact timing of onset and speed of progression of his Cushingoid features is difficult to determine. Due to his history of poor glycemic control, obese body habitus, and poor nutrition, his hypertension was initially suspected to be related to non-adherence to his treatment plan and poor diet. His headache was initially attributed to his hypertension since it resolved when his blood pressure normalized. 

During the initial diagnostic process, multiple disease processes were considered including Cushing syndrome and Mauriac syndrome. The table below compares our patient’s signs and symptoms with those of our leading differential diagnoses (Table 2).

**Table 2:** Leading differential diagnoses and their associations +Primary sign or symptom associated with the disease, present in our patient; ***Complication that can arise from the disease process, present in our patient; -Primary sign or symptom associated with the disease, not seen in our patient DM; diabetes mellitus; PPA: pediatric pituitary adenoma; DKA: diabetic ketoacidosis

Leading differential diagnoses and their associations					
	Type 1 DM	Cushing syndrome	ACTH-secreting PPA	Non-secreting PPA	Mauriac syndrome
DKA	+				***
Hyperglycemia	+	+	+		+
Moon facies		+	+		+
Truncal obesity		+	+		+
Dyslipidemia	***	***			+
Headache	***		+	+	
Hypertension	***	+			
Delayed growth	***	***			+
Delayed puberty	***	***			+
Transaminitis					+
Vision disturbance	***		***	***	
Weight gain	+	+	+		+
Galactorrhea			-		
Bruising/bleeding			-		
Polyuria	+		+		

While Cushing and Mauriac syndromes were considered after the patient initially presented with facial edema, it was not until his striae appeared and his hypertension was refractory to treatment that Cushing syndrome became our working diagnosis. Workup, including a midnight salivary cortisol or a dexamethasone suppression test, was not performed due to the patient’s lack of follow-up. Following admission, the primary team was planning to discuss further inpatient workup for Cushing syndrome with endocrinology the next day. Due to the sudden change in headache quality, an urgent CT head was obtained, which revealed the likely cause of the patient's signs and symptoms. As such, it was felt that a dexamethasone suppression test was no longer indicated.

Immediately following the discovery of the pituitary macroadenoma, multiple labs were obtained to evaluate the function of the hypothalamic-pituitary axis. Unfortunately, most of the pituitary hormones were not measured prior to presumed apoplexy, and hence the results are difficult to interpret. Serum total cortisol levels were elevated prior to presumed apoplexy and fell dramatically following apoplexy. As such, we can assume that the tumor was secreting ACTH prior to that time.

Once our patient reported a sudden change in the quality of his headache that he described as “the worst headache of his life,” a head CT without contrast was ordered. Had the diagnostic CT or preoperative MRI been obtained with contrast, it is likely that at least one of the studies would have shown an absence of blood flow to the pituitary region. As such, pituitary apoplexy is the likely cause of our patient’s severe, sudden-onset headache that ultimately led to his diagnosis.

As of 2011, there were only five reported cases of ACTH-secreting pituitary adenomas presenting with pituitary apoplexy. Most cases of pituitary apoplexy are associated with hemorrhage but, as evident in our patient, apoplexy can also be associated with infarction without hemorrhage. A literature review and case report published in September 2021 claims to present the 22nd patient with clinical pituitary apoplexy in patients younger than 20 years of age since 1980, when the CT was invented. At the time of this writing, our patient would be the 23rd patient with clinical pituitary apoplexy under the age of 20 years and would be the youngest patient with an ACTH-secreting pituitary adenoma to be reported [7].

## Conclusions

We described the case of an 11-year-old African American male, with poorly controlled T1DM, confirmed by the presence of GAD-65 and IA-2 antibodies. He initially presented with uncontrolled hyperglycemia and later developed Cushingoid features. He was found to have a functioning pituitary macroadenoma with subsequent apoplexy of the gland. Clinical pituitary apoplexy, with ACTH-secreting pituitary adenoma, is an extremely rare phenomenon, especially in pediatric patients, but should be considered in patients presenting with refractory hyperglycemia, hypertension, and other signs and symptoms of Cushing disease.
